# Selectivity Enhancement in Electronic Nose Based on an Optimized DQN

**DOI:** 10.3390/s17102356

**Published:** 2017-10-16

**Authors:** Yu Wang, Jianguo Xing, Shu Qian

**Affiliations:** School of Computer Science & Information Engineering, Zhejiang Gongshang University, Hangzhou 310018, China; 15060401010@pop.zjgsu.edu.cn (Y.W.); 16020000318@pop.zjgsu.edu.cn (S.Q.)

**Keywords:** e-nose, DQN, CNN, flow modulation

## Abstract

In order to enhance the selectivity of metal oxide gas sensors, we use a flow modulation method to exploit transient sensor information. The method is based on modulating the flow of the carrier gas that brings the species to be measured into the sensor chamber. We present an active perception strategy by using a DQN which can optimize the flow modulation online. The advantage of DQN is not only that the classification accuracy is higher than traditional methods such as PCA, but also that it has a good adaptability under small samples and labeled data. From observed values of the sensors array and its past experiences, the DQN learns an action policy to change the flow speed dynamically that maximizes the total rewards (or minimizes the classification error). Meanwhile, a CNN is trained to predict sample class and reward according to current actions and observation of sensors. We demonstrate our proposed methods on a gases classification problem in a real time environment. The results show that the DQN learns to modulate flow to classify different gas and the correct rates of gases are: sesame oil 100%, lactic acid 80%, acetaldehyde 80%, acetic acid 80%, and ethyl acetate 100%, the average correct rate is 88%. Compared with the traditional method, the results of PCA are: sesame oil 100%, acetic acid 24%, acetaldehyde 100%, lactic acid 56%, ethyl acetate 68%, the average accuracy rate is 69.6%. DQN uses fewer steps to achieve higher recognition accuracy and improve the recognition speed, and to reduce the training and testing costs.

## 1. Introduction

Electronic nose with metal oxide (MOX) gas sensor array is widely used in various fields [1,2,3,4,5,6,7,8] due to its simple structure, low cost and high sensitivity. Successful applications of electronic nose have been reported not only in the traditional food industry and environment monitoring, but also in medical applications such as cancer diagnosis. For example, Salvato et al. [9] proposed a holistic approach to the analysis of electronic nose generated olfactive patterns. This strategy allows for the simultaneous evaluation and combination of the informative contents provided by the two most common artificial olfaction approaches to volatile organic compounds (VOC) mixtures classification. Natale et al. [10] investigated the possibility of using an electronic nose to check whether volatile compounds present in expired air may diagnose lung cancer. Breath samples were collected and immediately analysed by an electronic nose.

However, a common challenge for MOX sensors or chemical sensors is their poor selectivity. There are two kinds of approaches to enhance selectivity of gas chemical sensors in general. One is to change the sensor’s working condition such as temperature modulation [11], gate bias modulation [12], which aims to result in a unique response pattern for each gas, thereby increasing the selectivity. Another way is to change the distribution of odorants around the sensor such as flow modulation [13], gasmodulation [14], e-mucosa [15], which exploit different diffusion and reaction velocity of the odorants to change their concentrations around sensors. 

Temperature modulation has been widely researched in recent years. For example, Vergara et al. [16] show how it is possible to optimize a multi-frequency signal to be used in the modulation of the operating temperature of an integrated gas sensor microarray. Huang et al. [17] investigated the gas sensing behavior of a single SnO_2_ gas sensor based on a dynamic measurement method. They used different heating waveform and frequency to modulate temperature. The results were compared with those of static measurement. Gosangi et al. [18] proposed a “pseudo sensor” method for changing the response characteristics of a sensor by dynamically modulating the heating temperature. They proposed an active sensing strategy based on partially observable Markov decision processes (POMDP) that allows the temperature modulation program to be optimized in real time, as the reactions of sensors to the environment. Describe active perception strategy as ternary classification problem, which uses the sensor model and Gaussian noise for simulation verification. 

In e-mucosa system [15], a sample first passes over a pre-concentrator employing a carbon black layer as the absorbent coating. These have been integrated with control electronics, a pre-concentrator, temperature control and a sample delivery system to produce a fully functional electronic nose (e-nose) instrument. Data from this device, when used with a pattern recognition method that utilizes temporal information and the large data set, the e-mucosa system improves the discrimination power of this instrument compared to conventional e-noses. One of the promising techniques is a microwave transduction technique [19,20], which is based on the change of electromagnetic properties of gas sensitive layer in the microwave range. Abdolrazzaghi et al. [21] developed a robust and fault-tolerant approach to microwave based sensitive measurements using Fuzzy Neural Network. A practical application of such method could be for high-cost industries such as biomedical/chemical wherein the accuracy of detection plays an important role. Rydosz et al. [22] used comb copolymer phthalocyanine (Pc) thin films as sensitive layers for microwave gas sensors at room temperature under exposure to various volatile organic compounds. The obtained results confirmed the possibility of using the microwave Pc-based sensors for exhaled acetone measurements. Mirsky [23] shows that the measurements in non-equilibrium conditions can reduce or even eliminate a relative contribution of interferences to a sensor signal.

While human beings and other animals use flow modulation routinely (sniff) for olfactory perception [24], there are very few reports about flow modulation in machine olfactory. Just as eye saccade in visual perception, animals make adjustments to sniff strength and duration in response to different olfactory tasks. The pioneering work of Mozell and colleagues [25,26] found that high-sorption rate odorant will induce a large response across olfactory mucosa when delivered at a high airflow and a smaller response when delivered at a lower airflow while low-sorption rate odorant show the opposite behavior. Contrary to temperature modulation, flow modulation has its bio-plausible. Barbri et al. [27] use flow modulation to obtain transient information and improve the selectivity of metal oxide gas sensors. The good results obtained which clearly outperform those obtained when the steady-state response used, prove the concept behind flow modulation. Ziyatdinov et al. [28] design an olfaction machine that could increase the lifetime and sensitivity of artificial chemo-sensory systems. They use an array of 16 metal-oxide gas sensors and combined with a chemical mechanical ventilator to simulate the biological respiration cycle. As a result, at early stages of measurement, such information is available which could make the technique suitable in early detection scenarios. However, neither of the aforementioned methods treats flow modulation as an active process nor proposes a systematic approach to optimizing flow speed online.

In this paper, we not only use flow modulation method to enhance selectivity of metal oxide sensors but also present an “active perception “strategy based on Deep Q Network (DQN) [29,30] that allows the gas flow to be optimized in real time, as the sensor reacts to a dynamic environment. We propose a combined DQN and Convolutional Neural Network (CNN) to fulfill this goal. DQN is an improved algorithm based on Q-Learning [31], using the deep learning network to solve the curse of dimensionality of large scale problem in practice. Without prior knowledge, DQN can be trained online for classification through observations and received rewards. The DQN learns an action policy to change the flow speed dynamically that maximizes the total rewards (or minimizes the classification error). Meanwhile, a CNN is trained to predict sample class and reward according to current actions and observation of sensors. We demonstrate our proposed methods on a gases classification problem in a real-time environment. The results show that the DQN learns to modulate flow to classify different gas. The results also show that the algorithm has high recognition accuracy for the five kinds of gases. The algorithm can improve the recognition speed of electronic nose and reduce the cost of training and testing.

## 2. DQN-CNN 

### 2.1. DQN

In a known environment, the dynamic sequence decision process is usually formalized into a Markov decision process (MDP); its characteristics are described by four tuple (S, A, P, R). In every step, agent based on current state $s_{t}$ and strategy π, select an action $a_{t}$ from action set A to execute. It will receive an instant reward $r_{t}$, then transit to a new state $s_{t+1}$. The goal of reinforcement learning is to find a strategy to maximize the expected discount reward.

(1) $$R_{t}=r_{t}+\gamma r_{t+1}+\gamma^{2}r_{t+2}+\cdots$$

where γ∈[0,1] is the discount factor, weight the importance of immediate and future rewards. In MDP, the optimal policy strategy can be calculated by value iterations [32].

Q-Learning is a model free reinforcement learning technique and states and rewards are generated by the environment. The purpose of Q-Learning is to find an optimal strategy to maximize total received reward. State is gotten from an observation function and in our electronic nose system state is the response value of the sensor array; a is the action that can change state, the action in the electronic nose system a is the flow rate. We use state-action value and Q value to estimate the value of an action in a given state under the optimal strategy, which is defined as follows:

(2) $$Q^{\pi }\left(s,a\right)=E[R_{t}|s_{t}=s,a_{t}=a,\pi ]$$

(3) $$V^{\pi }\left(s\right)=E_{a∼\pi \left(s\right)}\left[Q^{\pi }\left(s,a\right)\right]$$

The preceding state-action value function (Q function for short) can be computed recursively with dynamic programming.

(4) $$Q^{\pi }\left(s,a\right)=E_{s^{\prime }}[r+\gamma E_{a^{\prime }∼\pi \left(s^{\prime }\right)}[Q^{\pi }\left(s^{\prime },a^{\prime }\right)]|s,a,\pi ]$$

Define the optimal $Q^{*}\left(s,a\right)$ as:

(5) $$Q^{*}\left(s,a\right)=\max_{\pi }Q^{\pi }\left(s,a\right)$$

For given strategy, the optimal $V^{*}\left(s\right)$ is:

(6) $$V^{*}\left(s\right)=\max_{a}Q^{*}\left(s,a\right)$$

Thus, it also shows that the optimal Q function satisfies the Bellman equation:

(7) Q*(s,a)=Es′[r+γQ*a′max(s′,a′)|s,a]

We define state-dependent action function:

(8) $$A^{\pi }\left(s,a\right)=Q^{\pi }\left(s,a\right)-V^{\pi }\left(s\right)$$

Note that $E_{a∼\pi \left(s\right)}\left[A^{\pi }\left(s,a\right)\right]=0$. Intuitively, the value function V measures its quality in a particular state. The Q function measures the value of the particular action in this state. The dominant function subtracts the value of the state from the Q function and obtains a relative measure of the importance of each action.

When the state space is very large, a common skill is to use a function to approximate it. For example, DQN uses neural network parameter θ instead of Q (s, a; θ). A neural network with at least one nonlinear hidden layer and enough nodes can approximate any functions. To train the network, DQN optimizes the following loss function sequence in iterations:

(9) $$L_{i}\left(\theta_{i}\right)=E_{\left(s,a,r,s^{\prime }\right)∼U\left(D\right)}\left[({\left(y_{i}^{\mathrm{DQN}}-Q\left(s,a;\theta_{i}\right)\right)}^{2}\right]$$

Where yiDQN=r+γQa′max(s′,a′|θi−) represent the target value of an action in a given state. $\theta_{i}^{-}$ represent the parameter of the target network. You can try using standard Q-Learning to learn Q(s,a;θ) parameters online, but this method is not performing well in actual use. A key innovation is the use of gradient descent to update the parameters of the target network via iterations, which greatly improves the stability of the algorithm. Gradient update is:

(10) $$\nabla_{\theta_{i}}L_{i}\left(\theta_{i}\right)=E_{s,a,r,s^{\prime }}\left[\left(y_{i}^{\mathrm{DQN}}-Q\left(s,a;\theta_{i}\right)\right)\nabla_{\theta_{i}}Q\left(s,a;\theta_{i}\right)\right]$$

We call such an algorithm off-policy because the states and rewards are obtained through behavior strategies. Another key factor is experience replay [33]. During learning, agent accumulates experience from every iteration and stores it in a data set. When training the Q network, we only use the data from the stored data set, which are randomly sampled D times. The sequence of loss functions is as follows:

(11) $$L_{i}\left(\theta_{i}\right)=E_{\left(s,a,r,s^{\prime }\right)∼U\left(D\right)}\left[({\left(y_{i}^{\mathrm{DQN}}-Q\left(s,a;\theta_{i}\right)\right)}^{2}\right]$$

To overcome the small sample size and correlation between training samples, DQN uses experience replay to improve data efficiency by reusing empirical samples in multiple updates. What is important is that it reduces variance and uniform sampling in the replay buffer to reduce the correlation between the samples used in the update. References [29,30,31,32,33,34] have proved that this is an effective method. In [29], samples are obtained from successive video frames in the game. Compared to the simple reinforcement learning problem (such as maze), the sample is much more relevant. If there is no experience replay, the algorithm will basically do the gradient descent in the same direction for a continuous period of time, so it is impossible to directly calculate the gradient convergence at the same step size. Therefore, experience replay avoids the problem by randomly selecting some experience from a memory pool.

### 2.2. DQN-CNN

**Figure 1:**
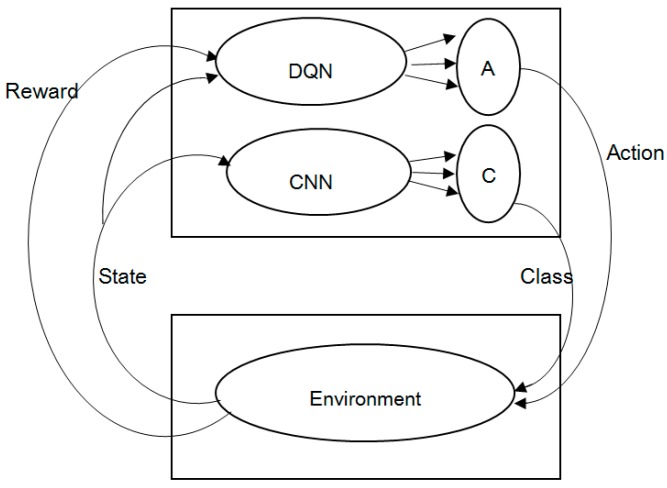
Optimized DQN (DQN-CNN) structure block diagram.

DQN will receive an immediate reward after selecting an action based on the optimal strategy. There are two kinds of rewards. If the action is correct, the reward is positive. Otherwise, it is negative. According to the conventional DQN [34], rewards are given by the game itself. In other words, DQN cannot determine the kind of rewards. The problem is that there is no such role in the electronic nose system that can determine the kind of rewards. So, in this paper, we proposed an optimized DQN, called DQN-CNN, the structure of the block diagram is shown in Figure 1. We use CNN as a role to approximate the rewards. CNN is best known for its ability to learn features invariant to translation, rotation and shifting without prior knowledge and human effort. In this research, we assume that when odorants pass through the surface of sensor array at different speeds, the sensors will exhibit some spatial invariant pattern which can be exploited. It is worthy to analyze the learned features in max pooling layers to see whether such invariances exist.

The inputs of DQN are the state values in the environment (e.g., the response value of the electronic nose sensor array) and rewards given by CNN. The output of DQN is the best action (different flow) according to the Q values at each episode. The best action can change the state of the environment, and different flow rates can change the sensor array responses. The inputs of CNN are the best action of DQN output and the state values in the environment. Besides, CNN is a three-layer convolutional neural network. The outputs are the perception classification base on train set labels and rewards. If the perception classification is correct, the reward is positive. Otherwise, it is negative.

### 2.3. DQN-CNN Algorithm

This approach has several advantages over standard online Q-learning. First, each step of experience is potentially used in many weight updates. Second, learning directly from consecutive samples is inefficient, due to the strong correlations between the samples; randomizing the samples breaks these correlations and therefore reduces the variance of the updates. Third, when learning on-policy the current parameters determine the next data sample that the parameters are trained on. We add CNN as a decision role. If the predicted result is consistent with the label, the reward is positive. Otherwise, it is negative. The full algorithm, which we call deep Q-learning, is presented in Algorithm 1.
**Algorithm 1** DQN-CNN with Experience Replay Initialize the memory stored in the experience of replay D, the number of iterations M Randomly initialize the Q-value function for iteration number = 1, M do randomly initialize the first action $a_{1}$ initialize the first state $s_{1}$ for t = 1, T do  if the probability is ϵ, select a random action $a_{t}$ otherwise select at=Q*a′max(st,a;θ) input $a_{t}$, $s_{t}$ into CNN, get classification $c_{t}=CNN\left(a_{t},s_{t}\right)$ if $c_{t}==\mathrm{label}$ then reward $r_{t}=1$ if t < T then reward $r_{t}=2$ else $r_{t}=0$ execute $a_{t}$, get $r_{t}$ and next state $s_{t+1}$ stored ($s_{t},a_{t},r_{t},s_{t+1}$) in D using a gradient descending of random small batches to get sample ($s_{j},a_{j},r_{j},s_{j+1}$)
yj={rjsj+1≠terminalrj+γQa′max(sj+1,a′;θ)sj+1=terminal Calculate the gradient of ${\left(y_{j}-Q\left(s_{j},a_{i};\theta \right)\right)}^{2}$ to update θ end if end for

## 3. Experiment 

### 3.1. Electronic Nose System 

**Figure 2:**
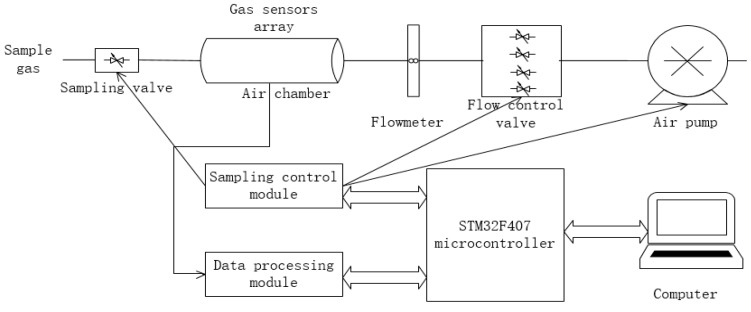
Structure diagram of E-nose.

**Figure 3:**
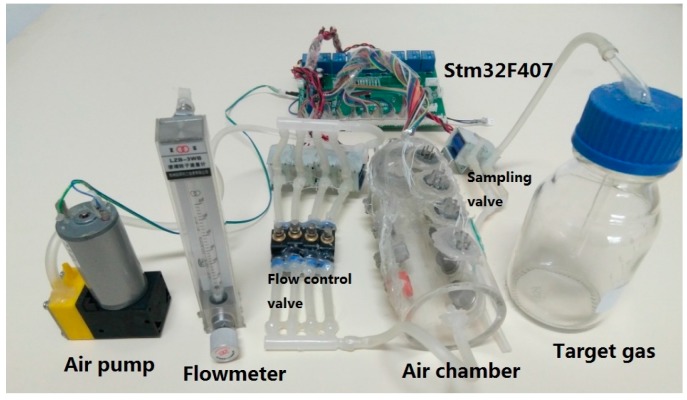
Image of the experimental setup.

The system is mainly composed of gas sensor array, sampling control module, data processing module and computer. The block diagram is shown in Figure 2. An image of the experimental setup is shown in Figure 3.

This paper uses 5 kinds of gases (namely, acetic acid, acetaldehyde, sesame oil, lactic acid and ethyl acetate) to validate the algorithm. The actions are 50 mL/min, 100 mL/min, 150 mL/min, 200 mL/min, 250 mL/min, 300 mL/min, No. 1–6. The raw data used in this paper are the actual response values measured at the above flow rates. In this experiment, the sensors we selected were produced by Wei Sheng Technology Co., Ltd., Zhengzhou, Henan province, China; the electronic nose system uses gas sensors, as shown in Table 1.

### 3.2. Experimental Analysis of 5 Kinds of Gases

**Figure 4:**
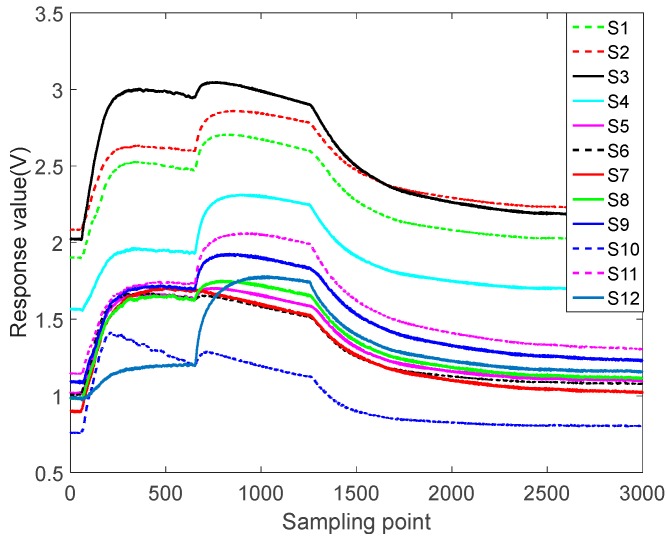
50 mL/min sesame oil raw data.

Figure 4 shows the raw data for sesame oil at 50 mL/min. The operation of sesame oil is the same as the other four samples. Sesame oil were taken 50 mL, placed in 250 mL cone bottle, sealed, static 1 h, so that the bottle reached saturation, the sensors preheat 1 h. Let 60 s of clean air in until the outputs of sensors are stable. Then test at different flow rates. At the end of the test, exhaust for 2.5 min until the corresponding sensors recovery baseline, and then do the next testing. Each sample was measured 25 times. We only do qualitative classification tests, not quantitative tests. According to the physical characteristics of the sensors, the minimum detectable gases concentrations are 300 ppm.

**Figure 5:**
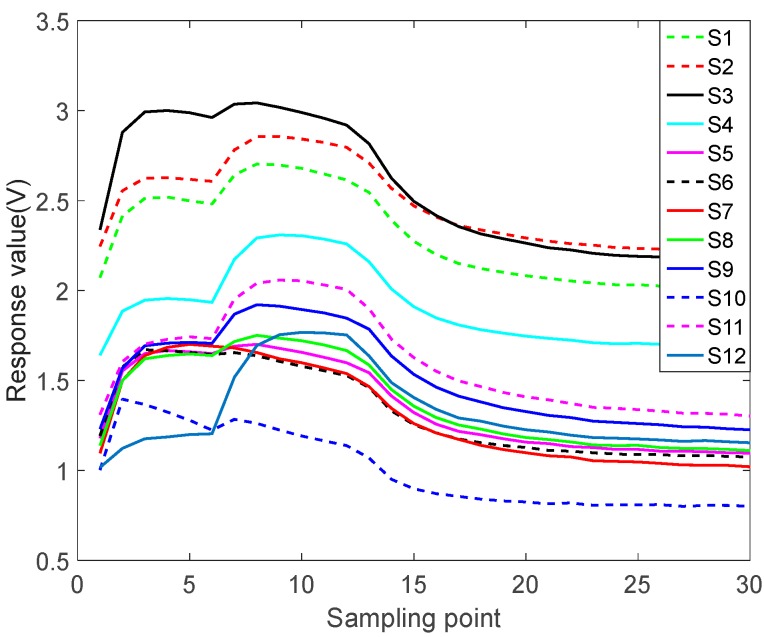
50 mL/min sesame oil feature extracted data.

The sampling frequency in the raw data is 10 Hz, in other word, sampling 10 times per second. Considering that the response values of the sensor array cannot change so much in a short time, and that the switching of the flow is delayed, it cannot respond immediately. This paper selects every 100 points on the raw data to extract feature, equivalent to sampling once per second, it can not only avoid the difference caused by the delay but also solve the problem of large amount of data. The data after feature extraction is shown in Figure 5.

As can be seen from Figure 5, the processed data size is 30 × 12. The continuous process is too complicated to simulate. In order to simplify the simulating process, this paper uses the response value of the same kind of gas at different actions, and makes a judgment every six steps. For example, a series of continuous actions are 5, 6, 5, 6, 3, 4, then extract the corresponding data from 250 mL/min, 300 mL/min, 250 mL/min, 300 mL/min, 150 mL/min, and 200 mL/min, respectively. Collect the corresponding sensor response values as the DQN datasets.

The datasets are divided into training sets and test sets. The training sets consist of five kinds of gases measured in six kinds of actions. The size is 6 × 150 × 12. The test sets have the same size, using the same measured method under the same conditions. The structure of DQN is two convolutional layers followed by three fully-connected layers. The first convolutional layer has 3 6 × 6 filters, the second has 6 3 × 3 filters. The first fully-connected layer has 540 units. The second fully-connected layer has 900 units. The third fully-connected layer has 150 units. The structure of CNN is three convolutional layers followed by two fully-connected layers. The first convolutional layer has 8 6 × 6 filters, the second 16 3 × 3 filters. The first fully-connected layer has 4608 units. The second fully-connected layer has 9216 units. The third fully-connected layer has 576 units. 

**Figure 6:**
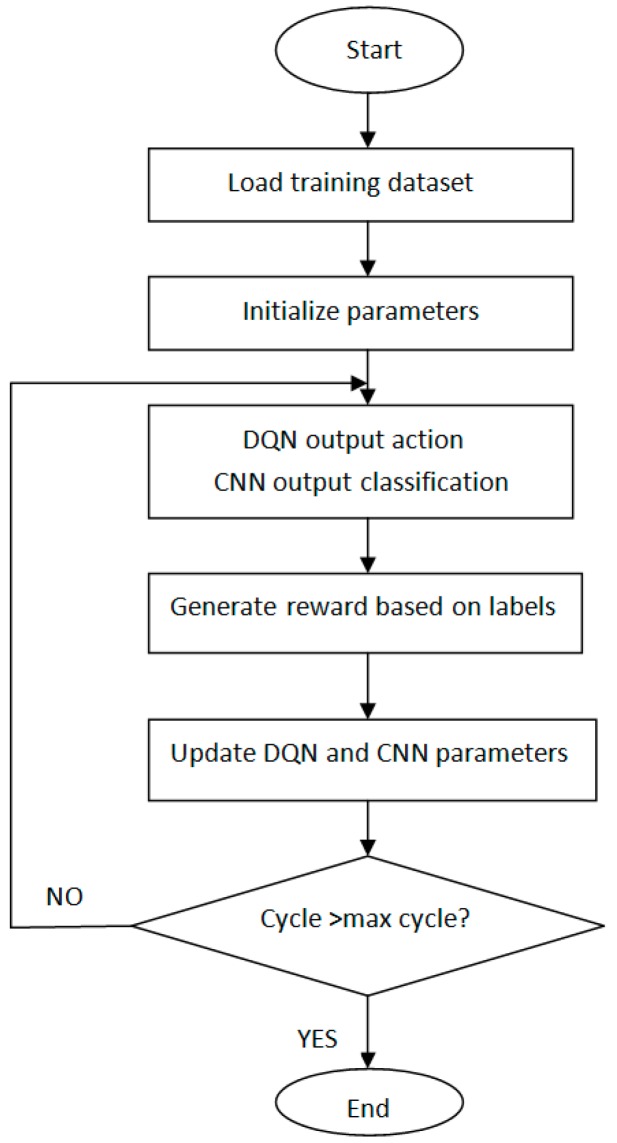
Flowchart of DQN-CNN.

We used one-hot encoding, so the numbers of output neurons are the same as the kinds of classification. What CNN outputs is not real class, but confidence level, a probability obtained by training. In the training phase, the rewards are determined by labels, and the rewards are used for training DQN. No reward was generated during the operative phase. According to the trained model, CNN uses the experience replay and off-policy to select a set of the most appropriate actions, and then outputs the predicted classification according to the response of sensors. A flowchart of DQN-CNN is given in Figure 6.

**Figure 7:**
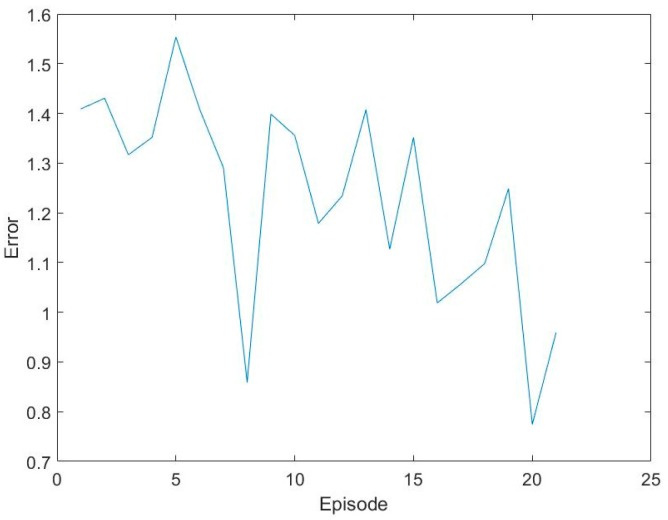
Training errors.

Input the training sets into the network for training and use the gradient descent method to update the weight of the DQN. The training error is shown in Figure 7.

As the number of training samples increases, training errors showed a downward trend. Due to the restrictions of electronic nose physical characteristics, less iteration would cause the final training errors larger. DQN selects the best action that can minimize difference based on the training data. The select action will change the response values of the sensor array. Meanwhile, DQN randomly selects whether to explore or experience. Explore means learning without experience replay. At the same time, the best action combined with the response values of current sensor array will be input into the CNN. According to the kinds of label, CNN will output the prediction classification and corresponding rewards in every episode.

**Figure 8:**
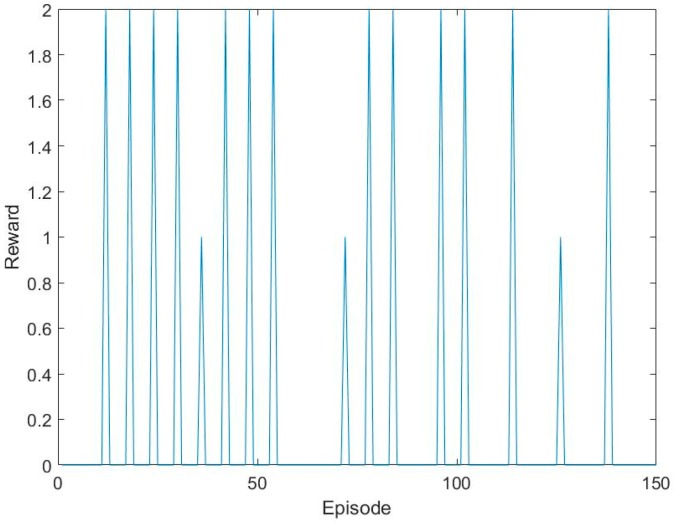
The reward of training sets.

In order to shorten the test time and improve efficiency, we decided to make a judgment at each of the six episodes. Repeat the above step six times. If the predicted steps are six, the reward is one. Besides, if the predicted steps are less than six, the reward is two, otherwise it is 0. The rewards of training sets are shown in Figure 8.

**Figure 9:**
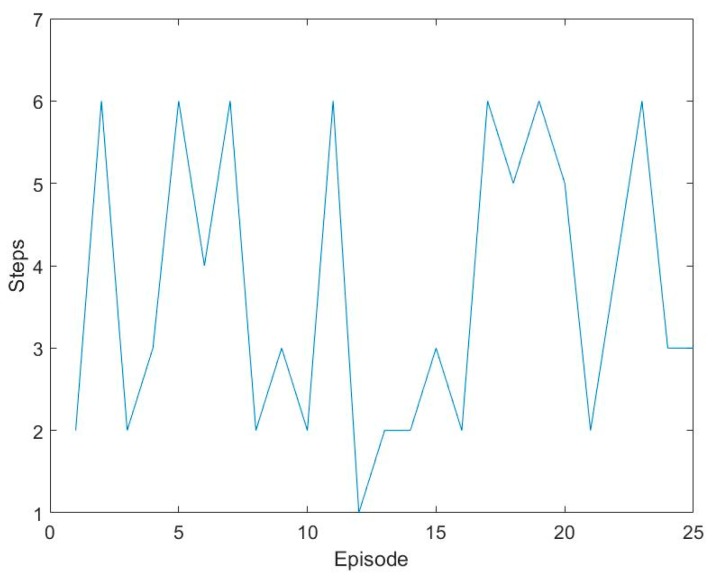
Number of steps.

Test the trained DQN-CNN. At each six steps, DQN makes a judgment, and then outputs the final predicted classification. The less the number of steps electronic nose required to predict, the faster the recognition speed is. Compared with the traditional electronic nose identify speed, the method used in this paper can greatly improve the identify speed. The steps used are shown in Figure 9.

We can see that a large part of the identify steps are less than six times, and often one time or two times. They can correctly identify the gas. Of course, the identify steps with six times contain the number that have not been identified. Table 2 is the numbers of identify steps.

The correct rates of the five gases are: 100% sesame oil, 80% lactic acid, 80% acetaldehyde, 80% acetic acid and 100% ethyl acetate, the average correct rate is 88%. DQN-CNN can achieve high correct rate through limited steps under the condition of less samples. Besides, DQN-CNN can not only improve the identify speed of electronic nose and reduce the training cost but also reduce the hardware and software cost.

### 3.3. Principal Component Analysis (PCA) 

**Figure 10:**
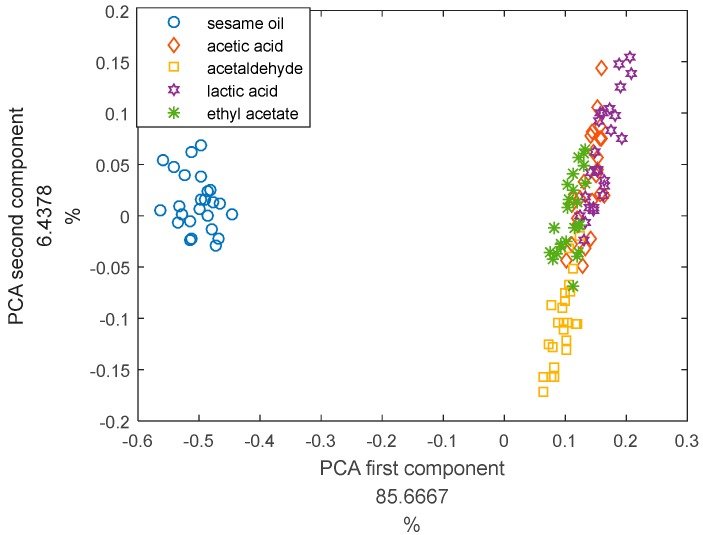
The result of principal component analysis.

To compare with the DQN, we used PCA to analyze the same experimental data. We use Euclidean distance for classification. In simple terms, we find the centers of the various kinds of training sets. Then, we calculate the distance between each point and the five central points in the test set. The nearest is the prediction classification. The feature used is the maximum value at steady state. The total sample number is 125. The number of training set is 100, and the number of test set is 25. We used 10-fold cross-validation to verify the correctness of the PCA algorithm. The first component of PCA is 85.7%. The second component of PCA is 6.4%. The results of PCA are: sesame oil 100%, acetic acid 24%, acetaldehyde 100%, lactic acid 56%, ethyl acetate 68%, the average accuracy rate is 69.6%. The advantage of DQN is not that it has a higher classification accuracy than traditional methods such as PCA, but that it has a good adaptability under small samples and labeled data. The result of principal component analysis is shown in Figure 10.

## 4. Conclusions

We propose an algorithm for active sensing of electronic nose pattern recognition based on DQN and CNN. The algorithm is based on the response values of sensor array, using the DQN to find a group action that maximizes the reward, using different flow to affect the response values of the sensor array. According to the response values of the sensor array, the experience replay of DQN outputs an action that maximizes the reward. The CNN output samples classification and rewards according to current actions and response values. The results show that the correct rate of five kinds of gases are: sesame oil 100%, lactic acid 80%, acetaldehyde 80%, acetic acid 80%, ethyl acetate 100%, the average accuracy rate is 88%. The results of PCA are: sesame oil 100%, acetic acid 24%, acetaldehyde 100%, lactic acid 56%, ethyl acetate 68%, and the average accuracy rate is 69.6%. The advantage of DQN is not that the classification accuracy is higher than traditional methods such as PCA, but that it has a good adaptability under small samples and labeled data. The algorithm can improve the identify speed of electronic nose and reduce the cost of training and testing.

## Figures and Tables

**Table 1:** Gas sensitive sensors list.

Number	Model	Nominal Test Target Gas
S1	MQ-8	hydrogen, coal, gas, etc.
S2	MQ-9B	carbon monoxide, etc.
S3	MQ-2	flammable gas, smoke, etc
S4	MQ-5	liquefied petroleum gas, methane, coal gas, etc
S5	MQ-135	ammonia, sulfides, etc.
S6	MQ-3B	alcohol, etc
S7	MQ-7B	carbon monoxide, etc.
S8	MQ-4	natural gas, methane, etc.
S9	MQ-2	flammable gas, smoke, etc.
S10	MQ-6	liquefied petroleum gas, isobutane, propane, etc.
S11	MQ-5	liquefied petroleum gas, methane, coal gas ,etc
S12	MQ-7	carbon monoxide, etc.

**Table 2:** Number of identify steps.

Steps	1	2	3	4	5	6
Sesame oil	0	2	1	0	0	2
Lactic acid	0	2	1	1	0	1
Acetaldehyde	1	2	0	0	0	2
Acetic acid	0	1	0	0	2	2
Ethyl acetate	0	1	2	1	0	1

## References

[1] Bieganowski A., Jaromin-Gleń K., Guz Ł., Łagńd G., Jozefaciuk G., Franus W., Suchorab Z., Sobczuk H. (2016). Evaluating Soil Moisture Status Using an e-Nose. Sensors.

[2] Altomare D.F., Porcelli F., Picciariello A., Pinto M., Di Lena M., Caputi lambrenghi O., Ugenti I., Guglielmi A., Vincenti L. (2016). The use of the PEN_3_ e-nose in the screening of colorectal cancer and polyps. Tech. Coloproctol..

[3] Li Q., Gu Y., Jia J. (2017). Classification of Multiple Chinese Liquors by Means of a QCM-based E-Nose and MDS-SVM Classifier. Sensors.

[4] Dai Y., Zhi R., Zhao L., Gao H., Shi B., Wang H. (2015). Longjing tea quality classification by fusion of features collected from E-nose. Chemom. Intell. Lab. Syst..

[5] Fu J., Li G., Qin Y., Freeman W.J. (2007). A pattern recognition method for electronic noses based on an olfactory neural network. Sens. Actuators B Chem..

[6] Jelen H.H., Wlazly K., Wasowicz E., Kaminski E. (1998). Solid-phase microextraction for the analysis of some alcohols and esters in beer: Comparison with static headspace method. J. Agric. Food Chem..

[7] Hong X., Wang J., Qi G. (2015). E-nose combined with chemometrics to trace tomato-juice quality. J. Food Eng..

[8] Ozmen A., Dogan E. (2009). Design of a Portable E-Nose Instrument for Gas Classifications. IEEE Trans. Instrum. Meas..

[9] Salvato M., Vito S.D., Esposito E., Massera E., Miglietta M., Fattoruso G., Francia G.D. (2016). An Holistic Approach to e-Nose Response Patterns Analysis—An Application to Nondestructive Tests. IEEE Sens. J..

[10] Di Natale C., Macagnano A., Martinelli E., Paolesse R., D’Arcangelo G., Roscioni C. (2003). Lung cancer identification by the analysis of breath by means of an array of non-selective gas sensors. Biosens. Bioelectron..

[11] Ortega A., Marco S., Perera A., Šundic T., Pardo A., Samitier J. (2001). An intelligent detector based on temperature modulation of a gas sensor with a digital signal processor. Sens. Actuators B Chem..

[12] Peng N., Zhang Q., Yi C.L., Tan O.K., Marzari N. (2008). Gate modulation in carbon nanotube field effect transistors-based NH_3_ gas sensors. Sens. Actuators B Chem..

[13] Bastuck M., Bur C., Spetz A.L., Andersson M., Schütze A. (2014). Gas identification based on bias induced hysteresis of a gas-sensitive SiC field effect transistor. J. Sens. Sens. Syst..

[14] Auerbach F. Pattern Recognition Using Gasmodulation. Proceedings of the 8th International Conference on Solid-State Sensors and Actuators 1995 and Eurosensors IX Transducers ’95.

[15] Harun F.K.C., Covington A., Gardner J.W. (2009). Portable e-Mucosa System: Mimicking the biological olfactory. Proced. Chem..

[16] Vergara A., Llobet E., Brezmes J., Ivanov P., Vilanova X., Gracia I., Cané C., Correig X. (2005). Optimised temperature modulation of metal oxide micro-hotplate gas sensors through multilevel pseudo random sequences. Sens. Actuators B Chem..

[17] Huang X., Meng F., Pi Z., Xu W., Liu J. (2004). Gas sensing behavior of a single tin dioxide sensor under dynamic temperature modulation. Sens. Actuators B Chem..

[18] Gosangi R., Gutierrez-Osuna R. (2010). Active Temperature Programming for Metal-Oxide Chemoresistors. IEEE Sens. J..

[19] Staszek K., Rydosz A., Maciak E., Wincza K., Gruszczynski S. (2017). Six-port microwave system for volatile organic compounds detection. Sens. Actuators B Chem..

[20] Zarifi M.H., Farsinezhad S., Abdolrazzaghi M., Daneshmand M., Shankar K. (2016). Selective microwave sensors exploiting the interaction of analytes with trap states in TiO_2_ nanotube arrays. Nanoscale.

[21] Abdolrazzaghi M., Zarifi M.H., Pedrycz W., Daneshmand M. (2016). Robust Ultra-High Resolution Microwave Planar Sensor Using Fuzzy Neural Network Approach. IEEE Sens. J..

[22] Rydosz A., Maciak E., Wincza K., Gruszczynski S. (2016). Microwave-based sensors with phthalocyanine films for acetone, ethanol and methanol detection. Sens. Actuators B Chem..

[23] Mirsky V.M. (2001). Affinity sensors in non-equilibrium conditions: Highly selective chemosensing by means of low selective chemosensors. Sensors.

[24] Joel M., Noam S. (2006). The Sniff is Part of the Olfactory Percept. Chem. Sens..

[25] Mozell M.M., Jagodowicz M. (1973). Chromatographic separation of odorants by the nose: Retention times measured across in vivo olfactory mucosa. Science.

[26] Youngentob S.L., Markert L.M., Hill T.W., Matyas E.P., Mozell M.M. (1991). Odorant identification in rats: An update. Physiol. Behav..

[27] Barbri N.E., Duran C., Brezmes J., Cañellas N., Ramírez J.L., Bouchikhi B., Llobet E. (2008). Selectivity Enhancement in Multisensor Systems Using Flow Modulation Techniques. Sensors.

[28] Ziyatdinov A., Fonollosa J., Fernández L., Gutierrez-Gálvez A., Marco S., Perera A. (2015). Bioinspired early detection through gas flow modulation in chemo-sensory systems. Sens. Actuators B Chem..

[29] Mnih V., Kavukcuoglu K., Silver D., Graves A., Antonoglou I., Wierstra D., Riedmiller M. (2013). Playing Atari with Deep Reinforcement Learning. arXiv.

[30] Wang Z., Schaul T., Hessel M., Van Hasselt H., Lanctot M., De Freitas N. (2015). Dueling Network Architectures for Deep Reinforcement Learning. arXiv.

[31] Sutton R.S., Barto A.G. (1998). Introduction to Reinforcement Learning.

[32] Watkins C.J.C.H. (1989). Learning from Delayed Rewards.

[33] Lin L. (1993). Reinforcement Learning for Robots Using Neural Networks. Ph.D. Thesis.

[34] Mnih V., Kavukcuoglu K., Silver D., Rusu A.A., Veness J., Bellemare M.G., Graves A., Riedmiller M., Fidjeland A.K., Ostrovski G. (2015). Human-level control through deep reinforcement learning. Nature.

